# Supplementary material for: A CAM-Related NF-YB Transcription Factor Enhances Multiple Abiotic Stress Tolerance in Arabidopsis
Source: Int J Mol Sci. 2024 Jun 28;25(13):7107. doi: 10.3390/ijms25137107 (PMC11241642; doi:10.3390/ijms25137107)
Supplement: Supplementary file 1 [file ijms-25-07107-s001.zip › ijms-3066416-supplementary.pdf]

```
>AT4G14540.1 | Symbols: NF-YB3 | "nuclear factor Y, subunit B3"
| chr4:8344349-8345324 FORWARD LENGTH=976
Length=976

 Score = 279 bits (308),  Expect = 4e-74
 Identities = 238/294 (81%), Gaps = 0/294 (0%)
 Strand=Plus/Plus

Query  47   CGACGAGGGAGCAGGACAGGCTGCTGCCGATAGCCAACGTGAGCAGGATCATGAAGAAGG  106
            ||||  | ||||| || ||| | || ||||| || ||||| ||||||||||||||||| |
Sbjct  367  CGACACGTGAGCAAGATAGGTTTCTACCGATCGCTAACGTTAGCAGGATCATGAAGAAAG  426

Query  107  CGCTGCCGGCGAACGCAAAGATATCGAAGGAGGCAAAGGAGACGGTGCAGGAGTGCGTTT  166
            | || || ||||||||||| || || ||||| || || || ||||| || ||||| || |
Sbjct  427  CACTTCCTGCGAACGCAAAAATCTCTAAGGATGCTAAAGAAACGGTTCAAGAGTGTGTAT  486

Query  167  CGGAGTTCATAAGCTTCATAACGGGGGAGGCGTCGGAGAAGTGCCAGAGGGAGAAGCGGA  226
            |||| |||||||| ||||| || || ||||| || || ||||| ||||| |||||| |||
Sbjct  487  CGGAATTCATAAGTTTCATCACCGGTGAGGCTTCTGACAAGTGTCAGAGAGAGAAGAGGA  546

Query  227  AGACGATCAACGGAGACGATCTGCTGTGGGCGATGACCACGCTGGGGTTCGAGGACTACG  286
            |||| |||||||| |||||||| || ||||||||||| ||||| ||||| ||||||||||
Sbjct  547  AGACAATCAACGGTGACGATCTTCTTTGGGCGATGACTACGCTAGGGTTTGAGGACTACG  606

Query  287  TGGAGCCTCTGAAGATATACTTGAGTAAGTTTAGGGAGATGGAGGGGGAGAAGA  340
            |||||||||| ||| | ||  ||   |||| ||||||| |||| || |||||||
Sbjct  607  TGGAGCCTCTCAAGGTTTATCTGCAAAAGTATAGGGAGGTGGAAGGAGAGAAGA  660
```

Figure S1. Pairwise sequence alignment between *KfNF-YB3* and its orthologue in *Arabidopsis thaliana* (Query: sequence of KfNF-YB3; Subject: sequence of orthologue *AtNF-YB3*.

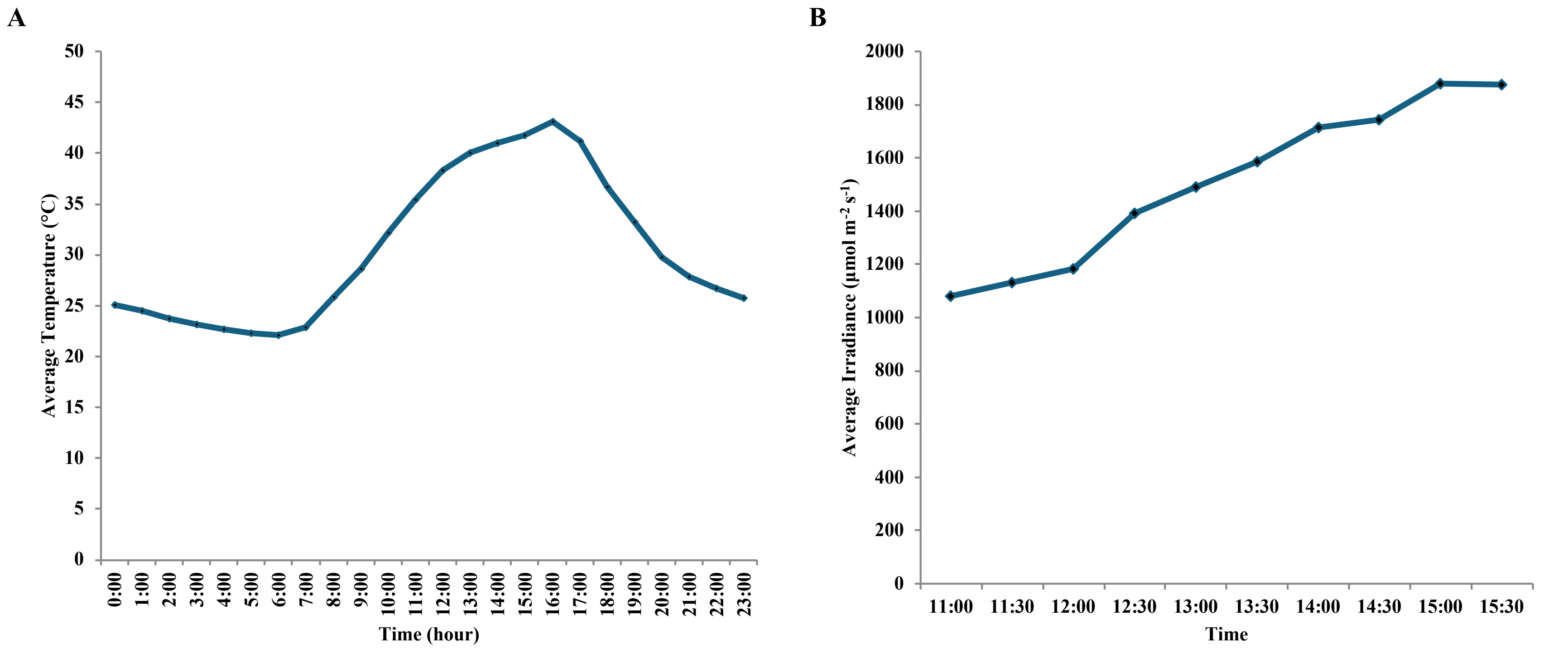

Figure S2. Daytime greenhouse conditions. (A) Average temperature over a 24-hour period. (B) Average daytime irradiance levels.
